# Cetuximab as first-line treatment for metastatic colorectal cancer (mCRC): a model-based economic evaluation in Indonesia setting

**DOI:** 10.1186/s12885-023-11253-y

**Published:** 2023-08-08

**Authors:** Septiara Putri, Siti Rizny F. Saldi, Levina Chandra Khoe, Ery Setiawan, Amila Megraini, Benjarin Santatiwongchai, Ryan R. Nugraha, Vetty Y. Permanasari, Mardiati Nadjib, Sudigdo Sastroasmoro, Armansyah Armansyah

**Affiliations:** 1 Health Policy and Administration Department, Faculty of Public Health, University of Indonesia, Depok, West Java 16424 Indonesia; 2 Center for Health Economics and Policy Studies (CHEPS), University of Indonesia, Depok, West Java 16424 Indonesia; 3 Center for Clinical Epidemiology and Evidence-Based Medicine (CEEBM), Cipto Mangunkusomo Hospital, Jakarta, 10430 Indonesia; 4 Department of Community Medicine, Faculty of Medicine, University of Indonesia, Jakarta, 10430 Indonesia; 5 Health Intervention and Technology Assessment Program (HITAP), Ministry of Public Health, Nonthaburi, 11000 Thailand; 6 Center for Population, Family, and Health, Department of Family Medicine and Population Health, University of Antwerp, Antwerp, 2610 Belgium; 7 Indonesian Health Technology Assessment Committee, Jakarta, 12950 Indonesia; 8 Department of Pediatrics, Faculty of Medicine, University of Indonesia, Jakarta, 10430 Indonesia; 9 Center for Health Financing and Insurance, Ministry of Health Republic of Indonesia, Jakarta, 12950 Indonesia

**Keywords:** Cetuximab, Colorectal cancer, Economic evaluation, Cost utility analysis

## Abstract

**Objectives:**

To assess the cost-effectiveness of cetuximab in combination with chemotherapy fluorouracil, oxaliplatin, and leucovorin (FOLFOX) or fluorouracil, irinotecan and leucovorin (FOLFIRI) compared to standard chemotherapy alone as a first-line treatment for metastatic colorectal cancer (mCRC) with positive KRAS wild type patients in Indonesia.

**Methods:**

A cost-utility analysis applying Markov model was constructed, with a societal perspective. Clinical evidence was derived from published clinical trials. Direct medical costs were gathered from hospital billings. Meanwhile, direct non-medical costs, indirect costs, and utility data were collected by directly interviewing patients. We applied 3% discount rate for both costs and outcomes. Probabilistic sensitivity analysis was performed to explore the model’s uncertainty. Additionally, using payer perspective, budget impact analysis was estimated to project the financial impact of treatment coverage.

**Results:**

There was no significant difference in life years gained (LYG) between cetuximab plus FOLFOX/FOLFIRI and chemotherapy alone. The incremental QALY was only one month, and the incremental cost-effectiveness ratio (ICER) was approximately IDR 3 billion/QALY for cetuximab plus chemotherapy. Using 1–3 GDP per capita (IDR 215 million or USD 14,350) as the current threshold, the cetuximab plus chemotherapy was not cost-effective. The budget impact analysis resulted that if cetuximab plus chemotherapy remain included in the benefits package under the Indonesian national health insurance (NHI) system, the payer would need more than IDR 1 trillion for five years.

**Conclusions:**

The combination of cetuximab and chemotherapy for mCRC is unlikely cost-effective and has a substantial financial impact on the system.

**Supplementary Information:**

The online version contains supplementary material available at 10.1186/s12885-023-11253-y.

## Background

Colorectal cancer (CRC) is one of the most prevalent cancers worldwide and continues to be a leading cause of mortality and morbidity. There were 1.8 million cases and 896,000 global deaths contributed by CRC in 2017 [1, 2]. Approximately 1.93 million new CRC cases are estimated in 2020, predicted to rise by 3.2 million in 2040 [3]. In Indonesia, as the third most common cancer, CRC incidence was relatively higher than in other Southeast Asia countries. There were 18,739 incident cases in 2017, and more than half of CRC patients were of productive and younger ages [1]. Lack of prevention programs such as screening and colonoscopy tests, as well as lifestyle changes are potentially contributing to this [4].

Patients may develop metastases that impact the critical survival rate during the disease course. For years, cytotoxic chemotherapy regimens such as FOLFOX (fluorouracil, oxaliplatin, and leucovorin) and FOLFIRI (fluorouracil, irinotecan, and leucovorin) have become standard treatment-mainly with palliative intent for metastatic colorectal cancer (mCRC) [5]. Over a decade, targeted therapy such as cetuximab (erbitux®), an IgG1 monoclonal antibody (mAb) against the epidermal growth factor receptor (EGFR) has been developed [6].

The CRYSTAL-phase III trial which evaluated 1198 patients demonstrated that cetuximab in combination with FOLFIRI was favourable compared with FOLFIRI alone [7]. In addition, the OPUS trial phase II provided similar findings when compared to FOLFOX-4 [8].Both of these trials reported that cetuximab provided the benefit of improving progression-free survival (PFS), particularly for patients with KRAS wild-type tumors [9]. From a published recent meta-analysis, compared to chemotherapy alone, cetuximab did not significantly improve both overall survival (OS) (HR = 0.99, 95% CI: 0.89–1.09, p = 0.78) and progression-free survival (PFS) (HR = 0.94, 95% CI: 0.81–1.10, p = 0.49) [10]. However, the overall response rate (ORR) did improve (RR = 1.34, 95% CI: 1.08–1.65, p = 0.00). For patients with a KRAS wild-type tumor, cetuximab provided an improvement in PFS (HR = 0.80, 95% CI: 0.65–0.99, p = 0.04) [10].

Cetuximab has been covered under the national health insurance scheme in Indonesia (namely as JKN, the payer agency is BPJS Kesehatan) since 2014. The national drug formulary stated that cetuximab should be used in combination with standard chemotherapy for confirmed KRAS wild-type (non-mutated) mCRC patients [11]. In 2017, cetuximab accounted for enormous total claims, approximately IDR 28.6 billion for 2.216 cases [12]. Due to the high total claimed costs reported by BPJS Kesehatan, drug price, and the substantial number of mCRC incidents, the Indonesia Health Technology Assessment mandated the university’s HTA team to evaluate the value for money of cetuximab. Therefore, this study aims to investigate the cost-effectiveness and financial impact of adding cetuximab to standard chemotherapy when compared to chemotherapy alone for mCRC patients with KRAS wild-type in Indonesia.

## Methods

### Target population

In this study, the target population of mCRC was patients (> 18 years old) with confirmed wild-type KRAS, without limitation of metastatic organ, gender, and race. A cancer diagnosis was confirmed following the criteria by National Comprehensive Cancer Network (NCCN) [13]. Patients included were only *de novo* patients, newly diagnosed that have not received any chemotherapy, radiation, and surgery. Regimen included:

1. Cetuximab + FOLFOX: Oxaliplatin 85 mg/m^2^ day 1; Leucovorin 400 mg/m^2^ day 1; 5-FU 400 mg/m^2^ day 1 continue with 1200 mg/m^2^ per day x 2 days. Cetuximab: weekly; first dose 400 mg/m^2^, further dose 250 mg/m^2^weekly; or per two weeks: first dose 400 mg/m^2^, a second dose and further 500 mg/m^2^ every week, maximum 12 cycles.
2. Cetuximab + FOLFIRI: Irinotecan 180 mg/m^2^ day 1; Leucovorin 400 mg/m2 day 1; 5-FU 400 mg/m2 day 1 continue with 1200 mg/m^2^ per day x 2 days ∙ Cetuximab: weekly; first dose 400 mg/m^2^, further dose 250 mg/m^2^ weekly; or per two-weeks: first dose 400 mg/m^2^, second dose and further 500 mg/m^2^ every week, maximum 12 cycles.

These eligibility criteria were applied to identify patients for the direct interviews in terms of costs and quality of life. The patient data were retrieved from four hospitals in Indonesia by exploring the medical records, drug utilization, and billing information.

### Model structure

**Fig. 1:**
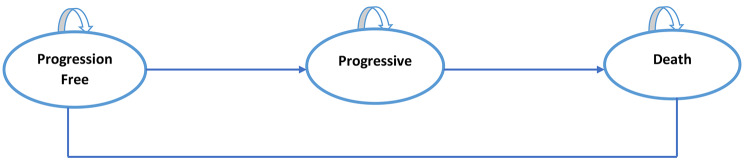
Schematic Markov model

The Markov model was constructed with three mutually exclusive states: progression-free, progressive, and death (Fig. 1. Schematic Markov model. Three states represented the disease course: progression-free, progressive, and death. It assumed that the patient can be in a progression-free state for some time and move to a progressive or death state. Furthermore, patients can remain in a progressive state or move to a death state. The progression-free state is defined as cancer having slightly developed or remains as a previous condition or non-significant tumor development exists. Furthermore, progressive is a condition where the cancer condition has developed, spread, and influenced other organs (usually confirmed by radiology).

In this model, we assumed that the patient received cetuximab in a very early stage and moved to or stayed in a progressive disease state until a terminal state. A lifetime horizon and 3-monthly cycle were applied. The duration was set after having a discussion with local experts (oncologists). Cancer patients are commonly assessed every three months, to understand whether they could transition to another health state.

In evaluating oncology drugs, the common modelling approaches are state transition models (e.g., Markov model) and partitioned survival models (PartSA) [14]. PartSA is gaining more popularity in the oncology field since the model reflects OS and PFS. Despite the recent development of the methods, our study, at the time of the study period, complied with the Indonesian HTA Guideline issued by the Indonesian HTA Committee (InaHTAC) [15]. The result of this study was used as informing decision-making at the national level and therefore, the methods should be in line with the national guideline.

Patient data were formally validated by an oncologist at the hospital to identify those meeting our inclusion criteria and fit the health states in the economic model. Oncologists at hospitals followed Response Evaluation Criteria in Solid Tumours (RECIST) criteria as a standard to help define patients’ conditions and fit them into model states [16]. Hence, although we used clinical evidence from published literature, this process was beneficial in choosing the eligible patients for cost and utility data.

### Clinical evidence and transition probabilities

We reviewed published systematic reviews of clinical trials and used a critical appraisal tool for systematic review by the Centre for Evidence-Based Medicine (CEBM), the University of Oxford to gather the efficacy of cetuximab in combination with FOLFOX/FOLFIRI. After reviewing published clinical trials, we constructed a network meta-analysis (NMA) to retrieve pooled estimates if there was no direct comparison between cetuximab plus chemotherapy and chemotherapy alone (including different chemotherapy regimens, i.e.: FOLFOX/FOLFIRI). We subsequently translated the clinical data into transition probabilities according to models’ cycles [17]. The details of the literature review and NMA were explained in detail in supplementary material 1. The NMA was performed using STATA MP13, and Microsoft Excel ® 2016 for the economic model.

### Costs

The cost-utility analysis was performed from a societal perspective. We estimated direct medical costs, direct non-medical costs and indirect costs in the analysis. Healthcare utilization related to treatment (admission, diagnostic test, laboratory test, drug, doctor’s visits, consultation, and hospitalization) and treatment complications were retrieved from patient medical records. Financial reports from the billing document (fiscal year 2018) were used to apply fees to each resource used, which allowed direct costs to be determined. Direct non-medical costs (travel costs, accommodation costs) and indirect costs (income lost) data were primarily collected by interviewing patients or caregivers when they visited a hospital through a structured questionnaire. These questionnaires were developed specifically for this study and administered by trained interviewers, with responses recorded in a standardized spreadsheet.

Travel expenses were calculated by multiplying the distance by the number of visits and the fuel price, which varied by transportation type. Accommodation costs were calculated based on the duration of use and cost of paid accommodation. Income lost or productivity losses were calculated by multiplying the time losses (number of visits) due to illness or during the treatment with the daily income rate. Self-reported wages for both patients and caregivers were recorded from the interview. If wages data were missing from the interview or respondents were unwilling to state their income, then the minimum standard wages at the provincial level were applied. Time losses of children and housework were not valued in this study.

Informed consent was explained by the data collector and signed by patients before the interview started. Mean costs were finally calculated according to health states in the Markov model. All costs were in the 2018 Indonesian Rupiah (IDR) value, we updated to 2023 value using Consumer Price Index (CPI) [18].

### Quality of life

Data for quality of life were gathered by interviewing patients, using the EQ-5D-5 L instrument that has been formally translated to the Indonesian language. This generic instrument has five dimensions with five specific problem levels for each dimension. The dimensions include mobility, self-care, usual activities, pain/discomfort, and anxiety/depression. Patients also completed a visual analog scale (VAS) as a part of this questionnaire. Quality of life scores was translated using the Indonesian EQ-5D-5 L value set [19].

### Incremental cost-effectiveness ratio (ICER)

The main result of this economic evaluation was represented as the incremental cost-effectiveness ratio (ICER), the ratio between incremental costs and incremental benefits. We expressed ICER as costs per quality-adjusted life years (QALYs). QALY was calculated by combining the length of life and quality of life value. Half-cycle correction and 3% discount rate were performed in this analysis. We used 1–3 GDP per capita (IDR 215 million) as a cost-effectiveness threshold, since Indonesia does not have a country-specific threshold yet. We followed all processes in economic evaluation according to the Indonesian National HTA guideline [15].

### Sensitivity analysis

To address the uncertainty of the health economic model, we performed deterministic sensitivity analysis (DSA) and probabilistic sensitivity analysis (PSA). PSA ran all model parameters with 5000 Monte-Carlo iterations simultaneously. The Cost-effectiveness acceptability curve (CEAC) was also presented to summarize the uncertainty from the cost-effectiveness estimate, with various ranges of acceptable thresholds.

### Budget impact analysis

Budget impact analysis (BIA) was undertaken to assess the financial consequences if cetuximab plus chemotherapy remains covered or is still on the benefit package under our NHI system. We simulated the scenario deterministically the financial impact using the payer perspective (BPJS Kesehatan) with a 5 years projection using recent epidemiological and claim data (see supplementary material 2). Two general scenarios were constructed: (1) If cetuximab and chemotherapy (either with FOLFOX or FOLFIRI) remained in the benefits package, and (2) if the payer only covered chemotherapy.

## Results

### Clinical outcomes

There were 13 systematic review or meta-analysis articles that reported cetuximab therapy for colorectal cancer. From these reviews, 4 studies were eligible for NMA [20–23]. The result is presented in Table 1. cetuximab + FOLFOX, cetuximab + FOLFIRI, and FOLFIRI were compared to FOLFOX. In terms of mortality, there was no significant difference between those interventions. However, the response rate shows a significant difference for cetuximab and chemotherapy compared to chemotherapy alone (Table 1). Transitional probabilities were derived from the systematic review and thus adjusted with the result from NMA results. We, therefore, translated the survival rate into monthly transitional probability [17]. The transition probabilities and other input parameters are summarized in Table 2.

**Table 1:** Clinical efficacy for each intervention

Intervention	Risk Ratio (CI 95%; p value)	
	Response rate	Mortality
FOLFIRI	RR 0.96 (CI95% 0.61–1.23; p = 0.790)	RR 1.10 (CI 95% 0.88–1.38; p 0.362)
Cetuximab + FOLFOX	RR 1.64 (CI95% 0.90–2.73; p = 0.001)	RR 0.95 (CI 95% 0.79–1.13; p 0.563)
Cetuximab + FOLFIRI	RR 0.96 (CI95% 0.80–2.31; p = 0.022)	RR 1.02 (CI 95% 0.81–1.29; p 0.823)

Reference: FOLFOX

**Table 2:** Input Parameter

Parameters	Value (Mean)	SE*	Distribution	Description	Reference
Transition probability					
tpStoS_folfox_1	0.555	0.111	Beta	transition probability remains in stable state (folfox)_month1-3	[21]
tpStoS_folfox_2	0.493	0.099	Beta	transition probability remains in stable state (folfox)_month4-6	[21]
tpStoS_folfox_3	0.410	0.082	Beta	transition probability remains in stable state (folfox)_month7-9	[21]
tpStoS_folfox_4	0.316	0.063	Beta	transition probability remains in stable state (folfox)_month10-12	[21]
tpStoD_year_1	0.005	0.001	Beta	transition probability from stable disease to death_year1	[24]
tpStoD_year_2	0.009	0.002	Beta	transition probability from stable disease to death_year2	[24]
tpStoD_year_3	0.015	0.003	Beta	transition probability from stable disease to death_year3	[24]
tpStoD_year_4	0.023	0.005	Beta	transition probability from stable disease to death_year4	[24]
tpStoD_year_5	0.038	0.008	Beta	transition probability from stable disease to death_year5	[24]
tpPtoD_folfox_1	0.023	0.005	Beta	transition probability from progressive to death (2nd line - cetux + folfiri as proxy)_month1-6	[21]
tpPtoD_folfox_2	0.031	0.006	Beta	transition probability from progressive to death (2nd line - cetux + folfiri as proxy)_month7-12	[21]
tpPtoD_folfox_3	0.056	0.011	Beta	transition probability from progressive to death (2nd line - cetux + folfiri as proxy)_month13-18	[21]
tpPtoD_folfox_4	0.075	0.015	Beta	transition probability from progressive to death (2nd line - cetux + folfiri as proxy)_month19-24	[21]
tpPtoD_folfox_5	0.040	0.008	Beta	transition probability from progressive to death (2nd line - cetux + folfiri as proxy)_month25-30	[21]
tpPtoD_folfox_6	0.037	0.007	Beta	transition probability from progressive to death (2nd line - cetux + folfiri as proxy)_month31-36	[21]
tpStoS_folfiri_1	0.569	0.114	Beta	transition probability remains in stable state (folfiri)_month1-3	[21] (adjusted with RR_folfiri)
tpStoS_folfiri_2	0.486	0.097	Beta	transition probability remains in stable state (folfiri)_month4-6	[21] (adjusted with RR_folfiri)
tpStoS_folfiri_3	0.406	0.081	Beta	transition probability remains in stable state (folfiri)_month7-9	[21] (adjusted with RR_folfiri)
tpStoS_folfiri_4	0.335	0.067	Beta	transition probability remains in stable state (folfiri)_month10-12	[21] (adjusted with RR_folfiri)
tpPtoD_folfiri_1	0.025	0.005	Beta	transition probability from progressive to death (2nd line - cetux + folfiri as proxy)_month1-6	[21] (adjusted with RR_folfiri)
tpPtoD_folfiri_2	0.034	0.007	Beta	transition probability from progressive to death (2nd line - cetux + folfiri as proxy)_month7-12	[21] (adjusted with RR_folfiri)
tpPtoD_folfiri_3	0.062	0.012	Beta	transition probability from progressive to death (2nd line - cetux + folfiri as proxy)_month13-18	[21] (adjusted with RR_folfiri)
tpPtoD_folfiri_4	0.083	0.017	Beta	transition probability from progressive to death (2nd line - cetux + folfiri as proxy)_month19-24	[21] (adjusted with RR_folfiri)
tpPtoD_folfiri_5	0.045	0.009	Beta	transition probability from progressive to death (2nd line - cetux + folfiri as proxy)_month25-30	[21] (adjusted with RR_folfiri)
tpPtoD_folfiri_6	0.040	0.008	Beta	transition probability from progressive to death (2nd line - cetux + folfiri as proxy)_month31-36	[21] (adjusted with RR_folfiri)
tpStoS_cetfolfox_1	0.761	0.152	Beta	transition probability remains in stable state (cetux + folfox)_month1-3	[21] (adjusted with RR_cetfolfox)
tpStoS_cetfolfox_2	0.678	0.136	Beta	transition probability remains in stable state (cetux + folfox)_month4-6	[21] (adjusted with RR_cetfolfox)
tpStoS_cetfolfox_3	0.589	0.118	Beta	transition probability remains in stable state (cetux + folfox)_month7-9	[21] (adjusted with RR_cetfolfox)
tpStoS_cetfolfox_4	0.501	0.100	Beta	transition probability remains in stable state (cetux + folfox)_month10-12	[21] (adjusted with RR_cetfolfox)
tpPtoD_cetfolfox_1	0.022	0.004	Beta	transition probability from progressive to death (2nd line - cetux + folfiri as proxy)_month1-6	[21] (adjusted with RR_cetfolfox)
tpPtoD_cetfolfox_2	0.029	0.006	Beta	transition probability from progressive to death (2nd line - cetux + folfiri as proxy)_month7-12	[21] (adjusted with RR_cetfolfox)
tpPtoD_cetfolfox_3	0.053	0.011	Beta	transition probability from progressive to death (2nd line - cetux + folfiri as proxy)_month13-18	[21] (adjusted with RR_cetfolfox)
tpPtoD_cetfolfox_4	0.071	0.014	Beta	transition probability from progressive to death (2nd line - cetux + folfiri as proxy)_month19-24	[21] (adjusted with RR_cetfolfox)
tpPtoD_cetfolfox_5	0.038	0.008	Beta	transition probability from progressive to death (2nd line - cetux + folfiri as proxy)_month25-30	[21] (adjusted with RR_cetfolfox)
tpPtoD_cetfolfox_6	0.035	0.007	Beta	transition probability from progressive to death (2nd line - cetux + folfiri as proxy)_month31-36	[21] (adjusted with RR_cetfolfox)
tpStoS_cetfolfiri_1	0.705	0.141	Beta	transition probability remains in stable state (cetux + folfiri)_month1-3	[21] (adjusted with RR_cetfolfiri)
tpStoS_cetfolfiri_2	0.619	0.124	Beta	transition probability remains in stable state (cetux + folfiri)_month4-6	[21] (adjusted with RR_cetfolfiri)
tpStoS_cetfolfiri_3	0.531	0.106	Beta	transition probability remains in stable state (cetux + folfiri)_month7-9	[21] (adjusted with RR_cetfolfiri)
tpStoS_cetfolfiri_4	0.446	0.089	Beta	transition probability remains in stable state (cetux + folfiri)_month10-12	[21] (adjusted with RR_cetfolfiri)
tpPtoD_cetfolfiri_1	0.024	0.005	Beta	transition probability from progressive to death (2nd line - cetux + folfiri as proxy)_month1-6	[21] (adjusted with RR_cetfolfiri)
tpPtoD_cetfolfiri_2	0.032	0.006	Beta	transition probability from progressive to death (2nd line - cetux + folfiri as proxy)_month7-12	[21] (adjusted with RR_cetfolfiri)
tpPtoD_cetfolfiri_3	0.057	0.011	Beta	transition probability from progressive to death (2nd line - cetux + folfiri as proxy)_month13-18	[21] (adjusted with RR_cetfolfiri)
tpPtoD_cetfolfiri_4	0.077	0.015	Beta	transition probability from progressive to death (2nd line - cetux + folfiri as proxy)_month19-24	[21] (adjusted with RR_cetfolfiri)
tpPtoD_cetfolfiri_5	0.041	0.008	Beta	transition probability from progressive to death (2nd line - cetux + folfiri as proxy)_month25-30	[21] (adjusted with RR_cetfolfiri)
tpPtoD_cetfolfiri_6	0.037	0.007	Beta	transition probability from progressive to death (2nd line - cetux + folfiri as proxy)_month31-36	[21] (adjusted with RR_cetfolfiri)
**Costs (IDR, 2023 value)**					
**Direct medical cost**					
CostDM_S_cetuxfolfiri	14,448,000	1,169,517	Gamma	direct medical cost related stable state (cetux + folfiri)	Hospital billing
CostDM_S_cetuxfolfox	12,613,787	1,050,322	Gamma	direct medical cost related stable state (cetux + folfox)	Hospital billing
CostDM_S_folfiri	14,448,000	1,169,517	Gamma	direct medical cost related stable state (folfiri)	Hospital billing
CostDM_S_folfox	12,613,787	1,050,322	Gamma	direct medical cost related stable state (folfox)	Hospital billing
CostDM_P_progressive	8,734,993	1,808,666	Gamma	direct medical cost related progressive state (2nd line - cetux + folfiri as proxy)	Hospital billing
**Direct non-medical cost**					
CostDnM_S_cetuxfolfiri	5,748,519	397,365	Gamma	direct non-medical cost related stable state (cetux + folfiri)	Interview
CostDnM_S_cetuxfolfox	5,748,519	397,365	Gamma	direct non-medical cost related stable state (cetux + folfox)	Interview
CostDnM_S_folfiri	5,748,519	397,365	Gamma	direct non-medical cost related stable state (folfiri)	Interview
CostDnM_S_folfox	5,748,519	397,365	Gamma	direct non-medical cost related stable state (folfox)	Interview
CostDnM_P_progressive	7,347,083	868,643	Gamma	direct non-medical cost related progressive state (2nd line - cetux + folfiri as proxy)	Interview
**Indirect cost**					
CostIn_S_cetuxfolfiri	6,149,008	903,267	Gamma	indirect medical cost related stable state (cetux + folfiri vs. folfiri)	Interview
CostIn_S_cetuxfolfox	6,149,008	903,267	Gamma	indirect medical cost related stable state (cetux + folfox vs. folfox)	Interview
CostIn_S_folfiri	6,149,008	903,267	Gamma	indirect medical cost related stable state (folfiri)	Interview
CostIn_S_folfox	6,149,008	903,267	Gamma	indirect medical cost related stable state (folfox)	Interview
CostIn_P_progressive	6,306,246	903,267	Gamma	indirect medical cost related progressive state (2nd line - cetux + folfiri as proxy)	Interview
**Cost of drug**					
Cetuximab	42,673,806	2,021,475	Gamma	drug cost for cetuximab	Hospital billing
Folfiri	9,589,710	876,419	Gamma	drug cost for folfiri	Hospital billing
Folfox	13,588,228	1,428,298	Gamma	drug cost for folfox	Hospital billing
**Discounting**					
D_cost	3%			discount rate for costs	HTA guideline [15]
D_outcome	3%			discount rate for benefit	HTA guideline [15]
**Utility**					
U_stable	0.798	0.031	Beta	utility related stable state	Interview
U_progressive	0.443	0.154	Beta	utility related progressive state	Interview
**Relative Risk**					
RR_cetuxfolfox_death	0.948	0.080	Lognormal	Relative risk cetux + folfox for death (folfox as reference)	Network MA
RR_cetuxfolfiri_death	1.026	0.108	Lognormal	Relative risk cetux + folfiri for death (folfox as reference)	Network MA
RR_folfiri_death	1.109	0.113	Lognormal	Relative risk folfiri for death (folfox as reference)	Network MA
RR_cetuxfolfox_resrate	1.644	0.211	Lognormal	Relative risk cetux + folfox for response rate (folfox as reference)	Network MA
RR_cetuxfolfiri_resrate	1.400	0.179	Lognormal	Relative risk cetux + folfiri for response rate (folfox as reference)	Network MA
RR_folfiri_resrate	0.965	0.114	Lognormal	Relative risk folfiri for response rate (folfox as reference)	Network MA

tp = transition probability, S = stable, P = progressive, D = death. Costs in IDR

*SE: standard error

### Costs and utility

Costs were derived from billing were reported in the input parameters Table 2. Direct non-medical costs and indirect costs from direct interviews (n = 22) are also estimated. There were no substantial differences in mean costs for cetuximab + FOLFOX and cetuximab + FOLFIRI, ranging between 12 and 14 million for the stable state, the progressive state has lower costs due to shorter hospitalization and patients received palliative care. The drug and chemotherapy costs are also relatively similar for both stable and progressive patients. The direct non-medical costs for stable patients were IDR 5.7 million, and IDR 7.3 million for progressive patients. Moreover, the productivity loss is approximately accounted for IDR 6 million. Total direct medical costs were driven by hospitalizations, while transportation and accommodation costs were contributed substantially to direct non-medical costs [25].

In terms of health-related quality of life, we initially interviewed 16 mCRC patients. However, 5 patients were excluded due to the incomplete medical record history that influenced the difficulties of medical status and defining disease state. Of 11 patients, 8 patients were in a stable state and only 3 were in a progressive state. If we use the Indonesian value set, the utility values for stable and progressive states were 0.798 and 0.443, respectively.

### Incremental cost-effectiveness ratio (ICER)

In the base case analysis, in terms of LYG, there was no significant difference between cetuximab plus chemotherapy versus chemotherapy alone, the incremental LYG was only around 2 months. Meanwhile, the incremental QALY was only 1 month. The highest ICER was cetuximab + FOLFIRI, approximately achieving USD IDR 3 billion/QALY. If we compare the current cost-effectiveness threshold to 1–3 GDP per capita (IDR 215 million, or USD 14,350), adding cetuximab to chemotherapy was not cost-effective. The summary of lifetime costs and QALY for each intervention is illustrated in Table 3. We estimated the ICER using a healthcare perspective, and the ICER was remain high about IDR 1.8 billion for cetuximab + FOLFOX, and IDR 3 billion for cetuximab + FOLFIRI (supplementary material 2).

**Table 3:** Cost-effectiveness results (societal perspective)

	FOLFOX	FOLFIRI^#^	Cetuximab + FOLFOX	Cetuximab + FOLFIRI
**Cost (IDR)**	629,695,618	575,092,934	**935,914,363**	880,419,546
**LY**	2.04	2.00	**2.18**	2.17
**QALY**	0.97	0.90	**1.07**	0.99
**ICER/LY**	1,529,891,385		**2,059,630,651**	1,797,316,762
**ICER/QALY**	769,921,617		**2,082,856,995**	3,385,521,116

Costs are in IDR, year 2023, #FOLFIRI = reference, societal perspective

### Sensitivity analysis

DSA results are visualized in supplementary material 2 in tornado diagram form. The most uncertain parameters include the utility in the progressive state and risk ratio from NMA. This is reflected in the limited number of patients for utility data in a progressive state. From this sensitivity analysis, the transition probability of the Cetuximab + FOLFIRI shows a high uncertainty-related mortality rate. This reflects the insignificant statistical results of OS from single studies which were incorporated in NMA.”

**Fig. 2:**
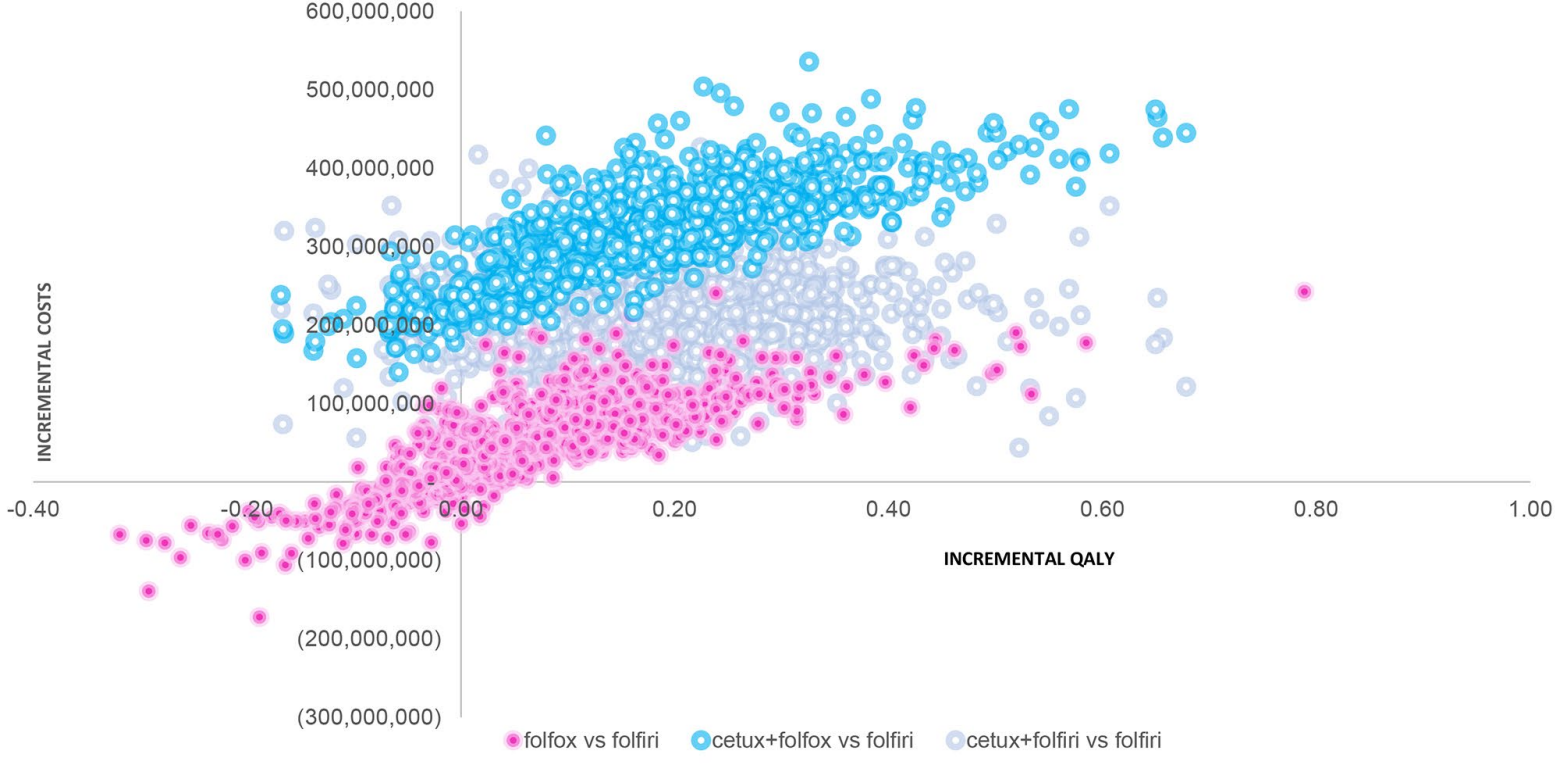
Cost-effectiveness plane

**Fig. 3:**
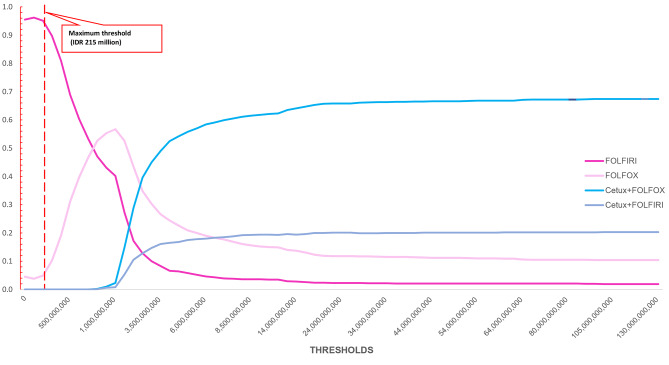
Cost-effectiveness acceptability curve (CEAC)

The cost-effectiveness plane plotted three possible interventions between cetuximab + FOLFOX, cetuximab + FOLFIRI, and only FOLFOX (with FOLFIRI as a reference). The plot showed that giving cetuximab with any FOLFOX/FOLFIRI indicated higher incremental costs and slight additional incremental QALY. Furthermore, there was no substantial probability to be cost-effective for cetuximab, less than 10% with a current maximum threshold. See Fig. 2. Cost-effectiveness plane. A distribution of incremental costs and QALYs based on 1000 Monte Carlo PSA simulation plotted in CE plane. The x and y-axis represent the difference in costs and QALYs, respectively. As plotted in the northeast quadrant, all interventions added costs and benefits compared to FOLFIRI. However, the costs were substantial relative to the small QALYs difference. and Fig. 3. Cost-effectiveness acceptability curve (CEAC). The graph indicates the probability of interventions compared to the FOLFIRI, with a range of threshold values. Using 1–3 GDP per capita, indicated that all intervention has low probability to be cost-effective.

### Budget impact analysis

For BIA, we focused on two main policy scenarios: (1) Patients who would be administered with Cetuximab plus standard chemotherapy (either FOLFOX or FOLFIRI) and (2) Patients who would be administered with chemotherapy alone (only FOLFOX or FOLFIRI). For mCRC patients with KRAS wild-type receiving chemotherapy alone, the financial impact for BPJS Kesehatan would be IDR 0.6 trillion for 5 years or about USD 40 million. On the other hand, if cetuximab plus chemotherapy remains provided, the payer would need IDR 1.3 trillion in five years.

**Fig. 4:**
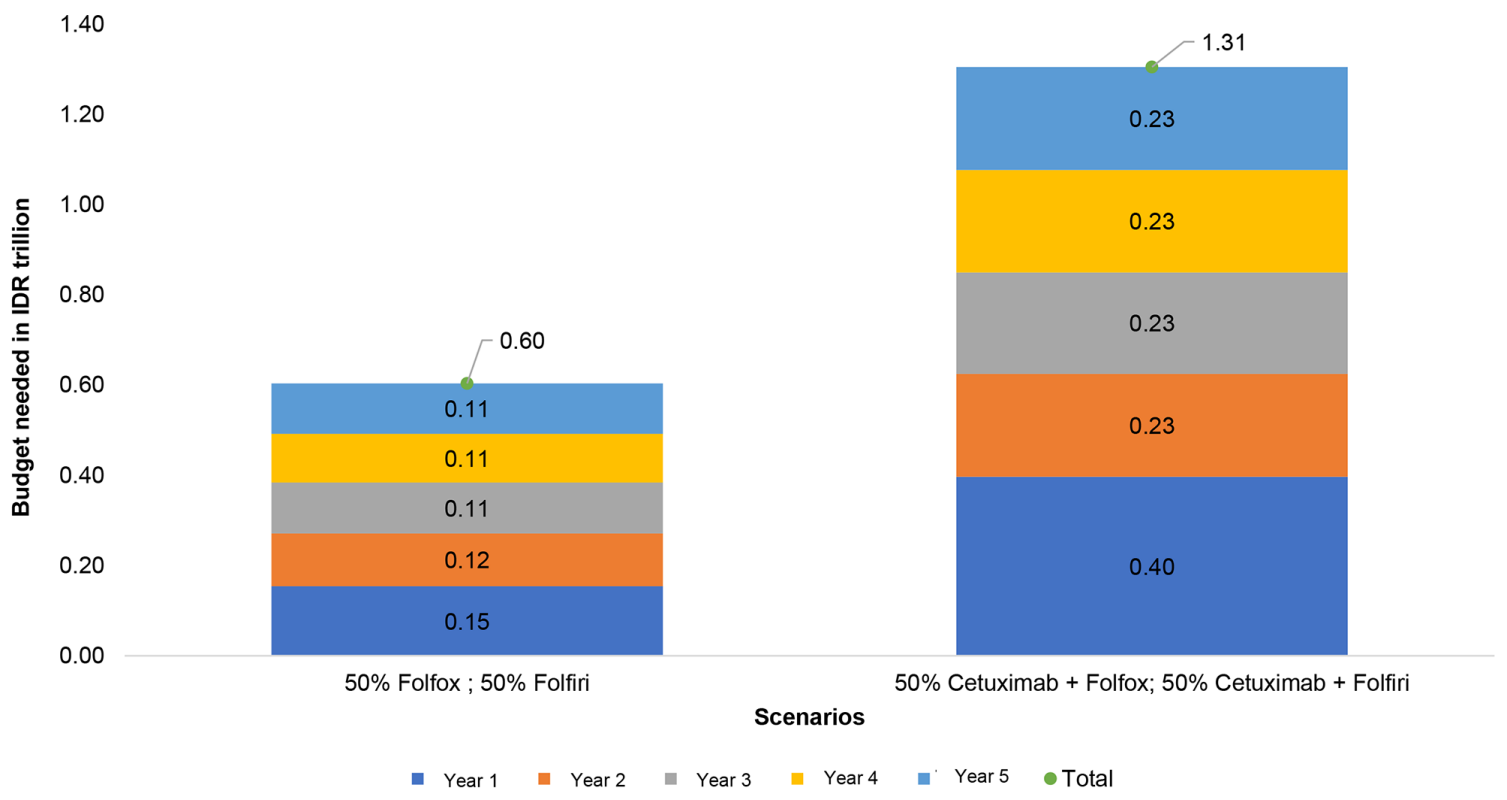
Budget impact analysis (BIA)

A transitional probability and dropout cases due to mortality had been considered in this analysis as referring to our constructed Markov model. However, this calculation used an assumption that all mCRC patients had received subsequent treatment at the hospital, while there might be a gap between actual patients and who truly access the hospital for treatment due to access barriers. This result provides substantial evidence for the payer in terms of the importance of price negotiation of the targeted therapy. The BIA result is illustrated in Fig. 4. Budget Impact Analysis (BIA). Financial impact estimation for 5 years, applying two scenarios: (1) NHI covers cetuximab plus chemotherapy (2) NHI covers chemotherapy only.

## Discussions

In Indonesia, cetuximab in combination with standard chemotherapy such as FOLFOX/FOLFIRI is not cost-effective. The expensive drug cost likely outweighs its benefit, the ICER exceeded the current acceptable threshold. If cetuximab and chemotherapy remain in the benefit package, the financial impact would be highly substantial as well. This result aligned with several economic evaluation studies, which suggest that the combination of chemotherapy and Cetuximab is not a cost-effective strategy compared with chemotherapy alone. A recent economic evaluation study reported that compared to FOLFOX-4 alone, cetuximab in combination with FOLFOX-4 was unlikely to be cost-effective in China [26]. The incremental QALY was only 0.15 within an increased cost of $19,079 The ICER was considerably high, $127,193/QALY while the threshold of willingness-to-pay in China is $27,934 [27]. Similarly, from a societal perspective in China, cetuximab plus chemotherapy compared to chemotherapy alone resulted in an ICER of US$ 164,044/QALY, exceeding the maximum threshold of US$ 28,106/QALY. A study by Shankaran et al. (2015) indicated that cetuximab as a first-line treatment for KRAS-positive WT patients was cost-effective if compared to another targeted therapy, bevacizumab [28].

Our current finding presents important evidence for decision-makers, particularly for the Ministry of Health and payer (BPJS Kesehatan), to re-evaluate the current benefit package on oncology therapies under NHI. This study demonstrated the representative economic model to estimate the long-term impact of adding cetuximab to chemotherapy for mCRC in the Indonesian setting, considering not only direct medical costs but also indirect costs from patients’ perspectives. We also collected primary data in terms of direct non-medical and indirect costs as well as utility, it allowed us to present the real costs and quality of life data. Despite the evidence for assessing value for money, we calculated the financial impact from the payer perspective, which is beneficial to informing decision-makers regarding affordability and resource allocation.

We have fully recognized the limitations of our study. First, the study had a limited number of samples, specifically for utility data due to the limited study period, resources constraints, and difficulties to include patients with confirmed KRAS testing-because, not all hospitals reported the KRAS status, or due to the KRAS testing were finished in other healthcare providers and not fully recorded in manual medical records. Second, our economic model used the assumption that cetuximab is used as first-line therapy, without considering different second-line targeted therapy. Besides, we assumed that all patients were compliant with treatment and chemotherapy cycles, although that may not be the case. Third, we did not define the metastases organ that may have a different clinical profile and be influenced by the benefit of the treatment. We gathered patients’ information with a common metastasis organ for colorectal cancer. Fourth, the models’ cycle was following our clinical assessment practice in Indonesia, applying a monthly cycle would be beneficial to ensure lesser error approximation and accurate probabilities conversion.

In addition, this study was conducted in 2016–2017 using the most available data at that time. Our NMA unlikely resulted from the high benefit of cetuximab in combination with chemotherapy for KRAS wild-type mCRC patients, trials showed the potential improvement of PFS only [25]. At the time of our study, we followed our national formularies policy questions and discussed with the clinical experts, revealing that tumor-sidedness was not considered as a criterion to receive cetuximab in Indonesia. We are fully aware that recently published studies with larger samples and considering the tumour location (left or right side) might confirm the favourable clinical outcome of this therapy. Nevertheless, our result is beneficial to support evidence-based policy-making for oncology drugs in NHI scheme 2018 in Indonesia [25]. [29–31].

Finally, our study indicates that adding cetuximab to standard chemotherapy for mCRC patients in Indonesia is not cost-effective. The financial impact is also considerably substantial for the NHI system. Decision makers must have careful consideration if cetuximab remains in the benefits package under NHI. In addition, there is also a need to explore and construct further discussions regarding the disinvestment policy in health technologies in Indonesia.

### Electronic supplementary material

Below is the link to the electronic supplementary material.

Supplementary Material 1

Supplementary Material 2

Supplementary Material 3

## Data Availability

The datasets (patient level) generated and/or analyzed during the current study are not publicly available due to data protection agreements under study sites/hospitals. Data are however available from the authors upon reasonable request and with permission of the Indonesian HTA committee Ministry of Health Republic of Indonesia via kptk.online@gmail.com.

## List of Abbreviations

FOLFOX: Fluorouracil, oxaliplatin, and leucovorin
FOLFIRI: Fluorouracil, irinotecan, and leucovorin
mCRC: Metastatic colorectal cancer
BPJS: Badan penyelenggara jaminan sosial (Indonesian health security agency)
DMC: Direct medical cost
DNMC: Direct non-medical cost
PFS: Progression-free survival
EFS: Event-free survival
OS: Overall survival
VAS: Visual analog scale
NMA: Network meta-analysis
ICER: Incremental cost-effectiveness ratio
LYG: Life years gained
QALY: Quality-adjusted life years
