# Supplementary material for: Cetuximab as first-line treatment for metastatic colorectal cancer (mCRC): a model-based economic evaluation in Indonesia setting
Source: BMC Cancer. 2023 Aug 8;23:731. doi: 10.1186/s12885-023-11253-y (PMC10408081; doi:10.1186/s12885-023-11253-y)
Supplement: Supplementary file 1 — Supplementary Material 1 [file 12885_2023_11253_MOESM1_ESM.docx]

**Supplementary Material 1. Network Meta-Analysis (NMA)**

**Table S1.1 Studies description for cetuximab plus chemotherapy compared to chemotherapy alone**

**Table S1.2 Clinical outcomes included in the network meta-analysis (NMA)**

**Figure S1.1 Schematic network meta-analysis (NMA)**

**Table S1.3 The result of network meta-analysis (NMA)**

**Technical details of NMA**

**Table S1.1 Studies description for cetuximab plus chemotherapy compared to chemotherapy alone**

| **Study ID** | **Bokemeyer et al. 2011** | | **Van Cutsem et al. 2015** | | **Colucci et al. 2005** | | | **Ocvirk et al. 2010** | | |
| --- | --- | --- | --- | --- | --- | --- | --- | --- | --- | --- |
| **Description** | **Cetuximab**  **+FOLFOX4** | **FOLFOX4** | **Cetuximab**  **+FOLFIRI** | **FOLFIRI** | | **FOLFIRI** | **FOLFOX4** | | **Cetuximab**  **+FOLFOX6** | **Cetuximab**  **+FOLFIRI** |
| **Number of subjects** | 82 | 97 | 316 | 350 | | 164 | 172 | | 34 | 28 |
| **Age, median (range)** | 62 (24-75) | 59 (36-82) | 61 (24-79) | 59 (19-84) | | 62(32-75) | 62 (31-75) | | 62.5 (55-67) | 64 (56-68) |
| **Gender** |  |  |  |  | |  |  | |  |  |
| Male, n (%) | 42 (51) | 55 (57) | 196 (62) | 211 (60.3) | | 93 (52) | 109 (60) | | 22 (65) | 17 (61) |
| Female, n (%) | 40 (49) | 42 (43) | 120 (38) | 139 (39.7) | | 85 (48) | 73 (40) | | 12 (35) | 11 (39) |
|  |  |  |  |  | |  |  | |  |  |
| **ECOG PS, at baseline, n(%)** |  |  |  |  | |  |  | |  |  |
| 0 | 32 (39) | 38 (39) | 183 (57.9) | 200 (57.1) | | 108 (60) | 106 (58) | | 20 (59) | 17 (61) |
| 1 | 44 (54) | 49 (51) | 120 (38.0) | 136 (38.9) | | 67 (38) | 68 (38) | | 14 (41) | 11 (39) |
| 2 | 6 (7) | 10 (10) | 13 (4.1) | 14 (4.0) | | 3 (2) | 8 (4) | |  |  |
|  |  |  |  |  | |  |  | |  |  |
| **Number of metastatic sites, n (%)** |  |  |  |  | |  |  | |  |  |
| ≤ 2 | 67 (82) | 75 (77) | 277 | 295 (84.3) | | 1:100 (56) | 1:99 (54) | | 28 (82) | 23 (82) |
| > 2 | 15 (18) | 22 (22) | 33 (10.4) | 49 (14.0) | | ≥ 2:78 (44) | ≥2:83 (46) | | 6 (18) | 5 (18) |
| Missing |  |  | 6 (1.9) | 6 (1.7) | |  |  | |  |  |
|  |  |  |  |  | |  |  | |  |  |
| **Duration of the treatment period, in months** | Med 5.7 months | Med 4.7 months | Med 7.41 months | Med 5.7 7 months | | Response: 9 (4-47) months;7,14 cycles | Response: 10 (5-27) months;7,26 cycles | |  |  |
|  |  |  |  |  | |  |  | |  |  |
| **Follow-up period, in months** | Follow-up  NR | Follow-up NR | Follow-up  NR | Follow-up NR | | 31(11-56) months | 31(11-56) months | | Patients’ follow-up was every 12 weeks until treatment ended or clinical cut-off date | |

**Table S1.2 Clinical outcomes included in the network meta-analysis (NMA)**

| **Study ID** | **Bokemeyer et al. 2011** | | **Van Cutsem et al. 2015** | | **Colucci et al. 2005** | | | **Ocvirk et al. 2010** | | |
| --- | --- | --- | --- | --- | --- | --- | --- | --- | --- | --- |
| **Description** | **Cetuximab**  **+FOLFOX4** | **FOLFOX4** | **Cetuximab**  **+FOLFIRI** | **FOLFIRI** | | **FOLFIRI** | **FOLFOX4** | | **Cetuximab**  **+FOLFOX6** | **Cetuximab**  **+FOLFIRI** |
| **Outcome name** | **Overall Survival (OS)** | | **Overall Survival (OS)** | | | **Overall Survival (OS)** | | | **Overall Survival (OS)** | |
| **Number of events (n)** | 55 | 71 |  |  | |  |  | | 21 | 16 |
| **OS median (IQR), months** | 22.8  (19.3-25.9) | 18.5  (16.4-22.6) | 23.5  (21.2-26.3) | 20.0  (17.4-21.7) | | 14 (1-28) | 15 (1-43) | | 22.5  (17.1-28.9) | 19.9  (11.9-na) |
| **Hazard Ratio for OS (95% CI)** | 0.855 (0.599-1.219) | | 0.80 (0.67-0.950 | | |  |  | |  |  |
|  |  | |  | | |  |  | |  |  |
| **Outcome name** | **Progression Free Survival (PFS)** | | **Progression Free Survival (PFS)** | | | **Progression Free Survival (PFS)** | | | **Progression Free Survival (PFS)** | |
| **Number of events (n)** |  |  |  |  | |  |  | | 26 | 20 |
| **PFS, median (IQR), months** | 8.3  (7.2-12.0) | 7.2  (5.6-7.4) | 9.9  (9.0-11.3) | 8.4  (7.4-9.2) | |  |  | | 9.1  (8.3-11.1) | 8.4 (3.2-11.3) |
| **Hazard Ratio for PFS (95% CI)** | 0.567  (0.375-0.856) | | 0.70  (0.56-0.87) | | |  |  | |  |  |
|  |  |  |  |  | |  |  | |  |  |
| **Outcome name** | **Response rate** | | **Response rate** | | | **Response rate** | | | **Response rate** | |
| **Number of events (n)** | 47 | 33 | 181 | 139 | | 56 | 62 | | 19 | 14 |
| **Number of patients (N)** | 82 | 97 | 316 | 350 | | 164 | 172 | | 34 | 28 |
| **ORR-ITT analysis (95% CI)** | 57 | 34 | 57.3 | 39.7 | | 31 | 34 | |  |  |
| **RR for response rate (95% CI)** | OR 2.551 (1.380-4.717) | | OR 2.07  (1.52-2.83) | | |  |  | | NA |  |

**Figure S1.1 Schematic network meta-analysis (NMA)**


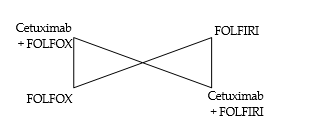


**Table S1.3 The result of network meta-analysis (NMA)**

| **Intervention** | **Relative Risk (95% CI; p value)** | |
| --- | --- | --- |
|  | **Response rate** | **Death** |
|  | **Reference: FOLFOX** | **Reference: FOLFOX** |
| **FOLFIRI** | RR 0.96 (95% CI 0.61 to 1.23; p =0.790) | RR 1.10 (95% CI 0.88 to 1.38; p =0.362) |
| **Cetuximab +FOLFOX** | RR 1.64 (95% CI 0.90 to 2.73; p =0.001) | RR 0.95 (95% CI 0.79 to 1.13; p =0.563) |
| **Cetuximab +FOLFIRI** | RR 1.40 (95% CI 0.80 to 2.31; p =0.022) | RR 1.02 (95% CI 0.81 to 1.29; p =0.823) |

Details of NMA:

We used a fixed effect model and tested the inconsistency test in to estimate indirect evidence with common chemotherapy comparators.

Defined name:

| **A (reference)** | FOLFOX |
| --- | --- |
| **B** | FOLFIRI |
| **C** | Cetuximab+FOLFOX |
| **D** | Cetuximab+FOLFIRI |

Note: indirect comparison AvsB and BvsC

Furthermore, the probabilities of each treatment are being tested (treatment being the best and other ranks), assuming the maximum parameter is the best. The targeted therapy in combination with chemotherapy indicated superiority compared to chemotherapy alone*.*

**Table S1.4 Network Rank**

| **Study id and rank** | **Treatment** | | | |
| --- | --- | --- | --- | --- |
|  | **FOLFOX** | **FOLFIRI** | **Cet+FOLFOX** | **Cet+FOLFIRI** |
| **Best** | 0.0 | 0.0 | 82.8 | 17.2 |
| **2^nd^** | 1.0 | 0.1 | 17.0 | 81.9 |
| **3^rd^** | 56.7 | 42.2 | 0.2 | 0.9 |
| **Worst** | 42.3 | 57.7 | 0.0 | 0.0 |

**Table S1.5 Multivariate meta-analysis (death)**

|  | exp(coef) | SE | z | P>\|z\| | 95% CI |  |  |
| --- | --- | --- | --- | --- | --- | --- | --- |
| _y_B |  |  |  |  |  |  |  |
| _cons | **1.109** | 0.126 | 0.12603 | 0.362 | 0.888 | 1.386 |  |
| _y_C |  |  |  |  |  |  |  |
| _cons | **0.948** | 0.087 | -0.58 | 0.563 | 0.792 | 1.135 |  |
| _y_D |  |  |  |  |  |  |  |
| _cons | **1.026** | 0.120 | 0.22 | 0.823 | 0.816 | 1.292 |  |

**Table S1.6 Multivariate meta-analysis (response rate)**

|  | exp(coef) | SE | z | P>\|z\| | 95% CI |  |  |
| --- | --- | --- | --- | --- | --- | --- | --- |
| _y_B |  |  |  |  |  |  |  |
| _cons | **0.965** | 0.129 | -0.270 | 0.790 | 0.742 | 1.254 |  |
| _y_C |  |  |  |  |  |  |  |
| _cons | **1.644** | 0.243 | 3.360 | 0.001 | 1.231 | 2.196 |  |
| _y_D |  |  |  |  |  |  |  |
| _cons | **1.400** | 0.206 | 2.280 | 0.022 | 1.049 | 1.868 |  |

From the results above, compared to the reference (A), treatment have favorable effect in response rate, but not death outcome.
