# Supplementary material for: Cetuximab as first-line treatment for metastatic colorectal cancer (mCRC): a model-based economic evaluation in Indonesia setting
Source: BMC Cancer. 2023 Aug 8;23:731. doi: 10.1186/s12885-023-11253-y (PMC10408081; doi:10.1186/s12885-023-11253-y)
Supplement: Supplementary file 3 — Supplementary Material 3 [file 12885_2023_11253_MOESM3_ESM.docx]

**Supplementary material 2. Sensitivity analysis**

**Table S2.1 Cost-effectiveness result using healthcare perspective**

**Figure S2.1 Deterministic sensitivity analysis (DSA)**

**a) FOLFOX**

**b) Cetuximab+FOLFOX**

**c) Cetuximab+FOLFIRI**

**Table S2.2 Baseline data for BIA**

**Table S2.1. Cost-effectiveness results (healthcare perspective)**

|  | **FOLFOX** | **FOLFIRI^#^** | **Cetuximab +FOLFOX** | **Cetuximab +FOLFIRI** |
| --- | --- | --- | --- | --- |
| **Cost (IDR)** | 310,659,568 | 271,469,881 | **596,030,389** | 553,676,012 |
| **LY** | 2.04 | 2.00 | **2.18** | 2.17 |
| **QALY** | 0.97 | 0.90 | **1.07** | 0.99 |
| **ICER /LY** | 1,098,040,622 |  | **1,852,647,088** | 1,661,217,170 |
| **ICER /QALY** | 552,591,654 |  | **1,873,539,289** | 3,129,156,712 |

**Figure S2.1 Deterministic sensitivity analysis (DSA)**

Data for both cost and utility were collected primarily from the patients by reporting the mean, standard deviation, standard error (in table 1) as well as confidence interval (CI). We also used CI information from our meta-analysis. These values were used to run the DSA. Furthermore, the DSA illustrated those variables uncertainty that related to ICER changes.

1. **Tornado diagram (FOLFOX)**

| **Parameter** | **SE** | **Lower value** | **Upper value** |
| --- | --- | --- | --- |
| U_progressive | 0.154 | 0.141 | 0.745 |
| tpPtoD_folfox_6 | 0.007 | 0.022 | 0.051 |
| tpPtoD_folfox_3 | 0.011 | 0.034 | 0.078 |
| tpPtoD_folfox_4 | 0.015 | 0.045 | 0.104 |
| tpStoS_folfox_1 | 0.005 | 0.014 | 0.032 |
| tpPtoD_folfox_2 | 0.006 | 0.019 | 0.043 |
| RR_folfiri_resrate | 0.114 | 0.740 | 1.250 |
| Folfox (drug cost) | 876,419 | 7,871,929 | 11,307,491 |
| Folfiri (drug cost) | 1,428,298 | 6,790,247 | 12,389,175 |
| CostDM_P_progressive | 1,808,666 | 6,044,727 | 13,134,697 |

**b) Tornado diagram (Cetuximab+FOLFOX)**

| **Parameter** | **SE** | **Lower value** | **Upper value** |
| --- | --- | --- | --- |
| RR_cetuxfolfox_death | 0.080 | 0.790 | 1.140 |
| tpStoS_cetfolfox_1 | 0.152 | 0.463 | 1.060 |
| U_progressive | 0.154 | 0.141 | 0.745 |
| tpPtoD_cetfolfox_6 | 0.007 | 0.021 | 0.048 |
| tpPtoD_cetfolfox_4 | 0.014 | 0.043 | 0.099 |
| tpPtoD_cetfolfox_3 | 0.011 | 0.032 | 0.074 |
| tpPtoD_cetfolfox_2 | 0.006 | 0.018 | 0.041 |
| RR_cetuxfolfox_resrate | 0.211 | 1.230 | 2.200 |
| RR_folfiri_resrate | 0.114 | 0.740 | 1.250 |
| tpStoS_cetfolfox_2 | 0.136 | 0.412 | 0.944 |

**c) Tornado diagram (Cetuximab+FOLFIRI)**

| **Parameter** | **SE** | **95% CI Lower** | **95% CI Upper** |
| --- | --- | --- | --- |
| U_progressive | 0.154 | 0.141 | 0.745 |
| tpStoS_cetfolfiri_2 | 0.124 | 0.376 | 0.861 |
| RR_cetuxfolfiri_death | 0.108 | 0.820 | 1.290 |
| RR_folfiri_death | 0.113 | 0.890 | 1.390 |
| tpPtoD_cetfolfiri_6 | 0.007 | 0.023 | 0.052 |
| tpStoS_cetfolfiri_1 | 0.141 | 0.428 | 0.981 |
| tpPtoD_cetfolfiri_4 | 0.015 | 0.047 | 0.107 |
| tpPtoD_cetfolfiri_3 | 0.011 | 0.035 | 0.080 |
| RR_cetuxfolfiri_resrate | 0.179 | 1.050 | 1.870 |
| tpPtoD_cetfolfiri_2 | 0.007 | 0.023 | 0.052 |

Note: X-axis refers to the changes in ICER, Y-axis refers to parameters incorporated in the health economic models. u=utility, tp=transition probability, Folfox/folfiri= drug costs), RR=relative risk.

The tornado diagrams illustrate the ten top uncertain parameters from the model. Parameters that are impactful for the model include relative risk (RR) and utility. As mentioned in clinical evidence, the published trial only shows the improvement in PFS, not another outcome, resulting in the high uncertainty for NMA results and the transition probability (due to the RR being adjusted in this parameter). The uncertainty of the utility parameter may be due to a limited sample of patients.

**Table S2.2 Baseline data for BIA**

| **Parameters** | **Values** | **Sources** |
| --- | --- | --- |
| **Total population** | 262,000,000 | Center of Statistic, 2017 |
| **Number of CRC patients** | 8,342 | BPJS Kesehatan, 2017 |
|  | 57,892 | GLOBOCAN, 2012 |
| **Incidence rate** | 14.2 per 100,000 | BPJS, 2017 |
|  | 12.8 per 100,000 | GLOBOCAN, 2012 |

We used the scenario using BPJS Kesehatan claim data because this study was intended to provide evidence for the NHI system in Indonesia.
